# 2-[4-(Tri­fluoro­meth­yl)phen­yl]-1*H*-benzimidazole

**DOI:** 10.1107/S1600536814012963

**Published:** 2014-06-11

**Authors:** M. S. Krishnamurthy, Noor Shahina Begum

**Affiliations:** a Department of Studies in Chemistry, Bangalore University, Bangalore 560 001, Karnataka, India

## Abstract

In the title compound, C_14_H_9_F_3_N_2_, the mean planes of the benzimidazole ring system and the tri­fluoro­methyl-substituted benzene ring form a dihedral angle of 30.1 (1)°. In the crystal, mol­ecules are linked by N—H⋯N hydrogen bonds into chains along [010]. Weak C—H⋯F hydrogen bonds and a weak C—H⋯π inter­action connect the chains into a two-dimensional network parallel to (001).

## Related literature   

For therapeutic and medicinal properties of benzimidazole derivatives, see: Ozden *et al.* (2004 ▶); Easman *et al.* (2001 ▶); Thakurdesai *et al.* (2007 ▶); Ansari & Lal (2009 ▶). For the bioactivity of fluorine-containing compounds, see: Ulrich (2004 ▶). For related structures, see: Jian *et al.* (2006 ▶); Rosepriya *et al.* (2011 ▶); Fathima *et al.* (2013 ▶); Krishnamurthy *et al.* (2013 ▶); Rashid *et al.* (2007 ▶).
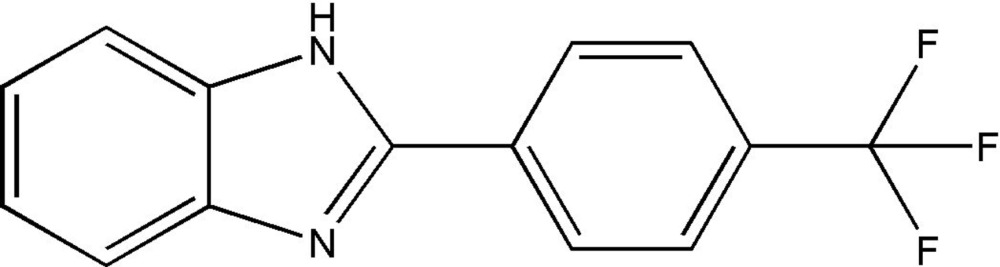


## Experimental   

### 

#### Crystal data  

C_14_H_9_F_3_N_2_
*M* *_r_* = 262.23
Orthorhombic, 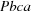

*a* = 9.2292 (9) Å
*b* = 9.8117 (10) Å
*c* = 25.347 (2) Å
*V* = 2295.2 (4) Å^3^
*Z* = 8
Mo *K*α radiation
μ = 0.13 mm^−1^
*T* = 296 K
0.18 × 0.16 × 0.16 mm

#### Data collection  

Bruker SMART APEX CCD detector diffractometer
Absorption correction: multi-scan (*SADABS*; Bruker, 1998 ▶) *T*_min_ = 0.978, *T*_max_ = 0.980
13079 measured reflections
2501 independent reflections
1671 reflections with *I* > 2σ(*I*)
*R*_int_ = 0.070

#### Refinement  

*R*[*F* ^2^ > 2σ(*F* ^2^)] = 0.056
*wR*(*F* ^2^) = 0.159
*S* = 0.90
2501 reflections
172 parameters
H-atom parameters constrained
Δρ_max_ = 0.55 e Å^−3^
Δρ_min_ = −0.33 e Å^−3^

### 

Data collection: *SMART* (Bruker, 1998 ▶); cell refinement: *SAINT-Plus* (Bruker, 1998 ▶); data reduction: *SAINT-Plus*; program(s) used to solve structure: *SHELXS97* (Sheldrick, 2008 ▶); program(s) used to refine structure: *SHELXL97* (Sheldrick, 2008 ▶); molecular graphics: *ORTEP-3 for Windows* (Farrugia, 2012 ▶) and *CAMERON* (Watkin *et al.*, 1996 ▶); software used to prepare material for publication: *WinGX* (Farrugia, 2012 ▶).

## Supplementary Material

Crystal structure: contains datablock(s) global, I. DOI: 10.1107/S1600536814012963/lh5706sup1.cif

Structure factors: contains datablock(s) I. DOI: 10.1107/S1600536814012963/lh5706Isup2.hkl

Supporting information file. DOI: 10.1107/S1600536814012963/lh5706Isup3.cml

CCDC reference: 1006709

Additional supporting information:  crystallographic information; 3D view; checkCIF report

## Figures and Tables

**Table 1:** Hydrogen-bond geometry (Å, °) *Cg* is the centroid of the N1/C5/C6/N2/C7 ring.

*D*—H⋯*A*	*D*—H	H⋯*A*	*D*⋯*A*	*D*—H⋯*A*
N1—H1⋯N2^i^	0.86	2.07	2.914 (3)	165
C12—H12⋯F1^ii^	0.93	2.57	3.374 (3)	144
C13—H13⋯F3^iii^	0.93	2.55	3.275 (4)	134
C2—H2⋯*Cg* ^iv^	0.93	2.94	3.700 (3)	140

Symmetry codes: (i) 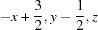; (ii) 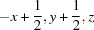; (iii) 
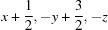
; (iv) 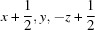.

**Fig. 1.**
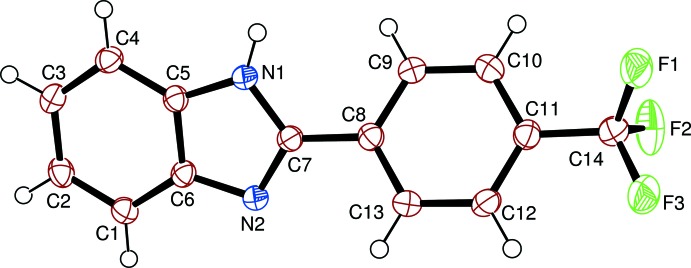
The molecular structure of the title compound with displacement ellipsoids drawn at the 50% probability level. H atoms are presented as small spheres of arbitrary radius.

**Fig. 2.**
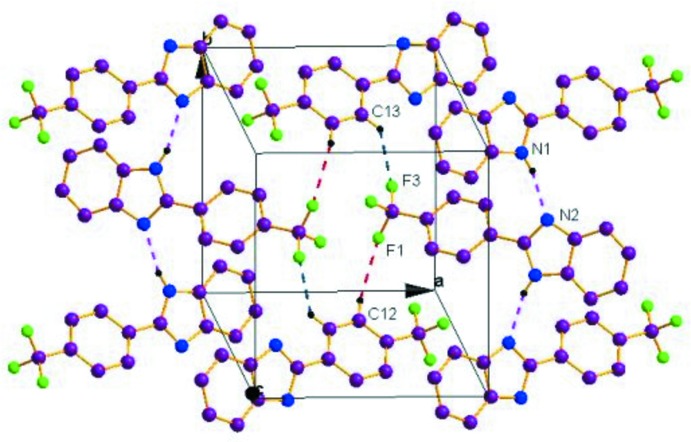
Part of the crystal structure showing hydrogen bonds with dotted lines. H-atoms not involved in hydrogen bonding have been excluded. The atoms labeled C13, N1 and C12 are related by the symmetry operators: -0.5+x, 1.5-y, -z; 1.5-x, 0.5+y, z and 0.5-x, -0.5+y, z, respectively.

## supplementary crystallographic information

### S1. Comment

Benzimidazole and its derivatives are regarded as a promising class of bio-active heterocyclic compounds that exhibit a range of biological activities such as antibacterial (Ozden *et al.*, 2004), anticancer (Easman *et al.*,2001), anti-HIV and anti-inflammatory (Ansari & Lal 2009; Thakurdesai *et al.*, 2007). In addition, compounds which contain fluorine have special bioactivity (Ulrich, 2004). The bond lengths and bond angles of the benzimidazole moiety in the title compound are in good agreement with those observed in related structures (Jian *et al.*, 2006; Rashid, *et al.*, 2007; Rosepriya *et al.*, 2011). The title compound is closely related to our previously reported compounds (Fathima *et al.*, 2013; Krishnamurthy *et al.*, 2013). The molecular structure of the title compound is shown in Fig. 1. The dihedral angle between the benzimidazole ring system and the trifluoro-substituted benzene ring is 30.1 (1)°. In the crystal structure, molecules are linked by N—H···N hydrogen bonds to form chains parallel to [010]. In addition, weak C—H···F hydrogen bonds and a weak C—H···π interaction connect chains into a two-dimensional network parallel to (001) (Fig. 2). The weak C—H···π interaction involves the centroid of the N1/C5/C6/N2/C7 ring (Table 1). In addition, the crystal packing involves the presence of short F···F contacts of 2.915 (3) Å.

### S2. Experimental

A mixture of 4-(trifluoromethyl)bezaldehyde (20 mmol, 0.35 g) and *o*-phenyldiamine (20 mmol, 0.22 g) in benzene (5.0 ml) was refluxed for 6 hrs on a water bath. The reaction mixture was cooled. The solid separated, was filtered and dried (yield: 0.38 g, 78% and m.p. 538 K). The title compound was dissolved in ethyl acetate and kept aside for slow evaporation to obtain pale yellow crystals suitable for X-ray diffraction studies.

### S3. Refinement

The H atoms were placed in calculated positions and refined in a riding-model approximation with C—H = 0.93 Å, N—H = 0.86 Å and with *U*_iso_(H) = 1.2*U*_eq_(N/C).

### Crystal data

**Table:**

C_14_H_9_F_3_N_2_	*F*(000) = 1072
*M**_r_* = 262.23	*D*_x_ = 1.518 Mg m^−^^3^
Orthorhombic, *P**b**c**a*	Mo *K*α radiation, λ = 0.71073 Å
Hall symbol: -P 2ac 2ab	Cell parameters from 1671 reflections
*a* = 9.2292 (9) Å	θ = 2.7–27.0°
*b* = 9.8117 (10) Å	µ = 0.13 mm^−^^1^
*c* = 25.347 (2) Å	*T* = 296 K
*V* = 2295.2 (4) Å^3^	Block, yellow
*Z* = 8	0.18 × 0.16 × 0.16 mm

### Data collection

**Table:**

Bruker SMART APEX CCD detector diffractometer	2501 independent reflections
Radiation source: fine-focus sealed tube	1671 reflections with *I* > 2σ(*I*)
Graphite monochromator	*R*_int_ = 0.070
ω scans	θ_max_ = 27.0°, θ_min_ = 2.7°
Absorption correction: multi-scan (*SADABS*; Bruker, 1998)	*h* = −11→11
*T*_min_ = 0.978, *T*_max_ = 0.980	*k* = −12→12
13079 measured reflections	*l* = −32→23

### Refinement

**Table:**

Refinement on *F*^2^	Primary atom site location: structure-invariant direct methods
Least-squares matrix: full	Secondary atom site location: difference Fourier map
*R*[*F*^2^ > 2σ(*F*^2^)] = 0.056	Hydrogen site location: inferred from neighbouring sites
*wR*(*F*^2^) = 0.159	H-atom parameters constrained
*S* = 0.90	*w* = 1/[σ^2^(*F*_o_^2^) + (0.0847*P*)^2^ + 2.1194*P*] where *P* = (*F*_o_^2^ + 2*F*_c_^2^)/3
2501 reflections	(Δ/σ)_max_ < 0.001
172 parameters	Δρ_max_ = 0.55 e Å^−^^3^
0 restraints	Δρ_min_ = −0.33 e Å^−^^3^

### Special details

**Table:**

Geometry. All s.u.'s (except the s.u. in the dihedral angle between two l.s. planes) are estimated using the full covariance matrix. The cell s.u.'s are taken into account individually in the estimation of s.u.'s in distances, angles and torsion angles; correlations between s.u.'s in cell parameters are only used when they are defined by crystal symmetry. An approximate (isotropic) treatment of cell s.u.'s is used for estimating s.u.'s involving l.s. planes.
Refinement. Refinement of *F*^2^ against ALL reflections. The weighted *R*-factor *wR* and goodness of fit *S* are based on *F*^2^, conventional *R*-factors *R* are based on *F*, with *F* set to zero for negative *F*^2^. The threshold expression of *F*^2^ > 2σ(*F*^2^) is used only for calculating *R*-factors(gt) *etc*. and is not relevant to the choice of reflections for refinement. *R*-factors based on *F*^2^ are statistically about twice as large as those based on *F*, and *R*- factors based on ALL data will be even larger.

### Fractional atomic coordinates and isotropic or equivalent isotropic displacement parameters (Å^2^)

**Table:**

	*x*	*y*	*z*	*U*_iso_*/*U*_eq_	
N1	0.7803 (2)	0.34060 (19)	−0.15282 (8)	0.0231 (5)	
H1	0.7333	0.2656	−0.1490	0.028*	
N2	0.8319 (2)	0.56324 (19)	−0.14790 (8)	0.0226 (5)	
F1	0.13831 (19)	0.43903 (16)	−0.01444 (8)	0.0575 (6)	
F2	0.09362 (17)	0.6088 (2)	−0.06475 (7)	0.0547 (6)	
F3	0.20033 (18)	0.63865 (18)	0.00860 (7)	0.0495 (5)	
C1	1.0744 (3)	0.5460 (3)	−0.19449 (10)	0.0266 (6)	
H1A	1.0979	0.6380	−0.1922	0.032*	
C2	1.1659 (3)	0.4549 (3)	−0.21894 (10)	0.0282 (6)	
H2	1.2515	0.4866	−0.2339	0.034*	
C3	1.1332 (3)	0.3152 (2)	−0.22181 (9)	0.0271 (6)	
H3	1.1975	0.2567	−0.2387	0.033*	
C4	1.0077 (3)	0.2629 (2)	−0.20016 (9)	0.0251 (5)	
H4	0.9867	0.1703	−0.2014	0.030*	
C5	0.9137 (2)	0.3561 (2)	−0.17622 (9)	0.0221 (5)	
C6	0.9454 (2)	0.4960 (2)	−0.17332 (9)	0.0225 (5)	
C7	0.7369 (2)	0.4661 (2)	−0.13685 (9)	0.0220 (5)	
C8	0.5975 (2)	0.4891 (2)	−0.11017 (9)	0.0229 (5)	
C9	0.4787 (3)	0.4059 (3)	−0.12079 (10)	0.0303 (6)	
H9	0.4874	0.3348	−0.1449	0.036*	
C10	0.3482 (3)	0.4286 (3)	−0.09573 (11)	0.0316 (6)	
H10	0.2691	0.3729	−0.1029	0.038*	
C11	0.3353 (3)	0.5344 (2)	−0.05983 (10)	0.0261 (6)	
C12	0.4518 (3)	0.6180 (2)	−0.04903 (9)	0.0274 (6)	
H12	0.4422	0.6892	−0.0251	0.033*	
C13	0.5836 (3)	0.5953 (2)	−0.07410 (9)	0.0252 (5)	
H13	0.6625	0.6512	−0.0668	0.030*	
C14	0.1920 (3)	0.5553 (3)	−0.03327 (10)	0.0292 (6)	

### Atomic displacement parameters (Å^2^)

**Table:**

	*U*^11^	*U*^22^	*U*^33^	*U*^12^	*U*^13^	*U*^23^
N1	0.0221 (10)	0.0181 (10)	0.0291 (11)	−0.0002 (8)	0.0020 (8)	0.0001 (8)
N2	0.0218 (10)	0.0200 (10)	0.0261 (11)	−0.0003 (8)	0.0011 (8)	−0.0008 (8)
F1	0.0508 (11)	0.0331 (10)	0.0887 (15)	−0.0011 (8)	0.0391 (10)	0.0039 (9)
F2	0.0301 (9)	0.0918 (15)	0.0422 (10)	0.0208 (9)	0.0039 (7)	0.0117 (9)
F3	0.0369 (10)	0.0623 (12)	0.0493 (10)	−0.0021 (8)	0.0102 (7)	−0.0253 (8)
C1	0.0237 (12)	0.0237 (13)	0.0325 (14)	−0.0018 (10)	0.0010 (10)	0.0015 (10)
C2	0.0226 (12)	0.0305 (14)	0.0315 (14)	−0.0013 (10)	0.0050 (10)	0.0051 (11)
C3	0.0264 (13)	0.0268 (13)	0.0283 (13)	0.0057 (10)	0.0013 (10)	−0.0019 (10)
C4	0.0268 (12)	0.0210 (12)	0.0275 (13)	0.0021 (10)	−0.0014 (10)	0.0004 (9)
C5	0.0204 (12)	0.0231 (12)	0.0228 (12)	0.0015 (9)	−0.0018 (9)	0.0009 (9)
C6	0.0220 (12)	0.0224 (12)	0.0230 (12)	0.0036 (9)	−0.0025 (9)	0.0005 (9)
C7	0.0215 (12)	0.0220 (12)	0.0226 (12)	0.0017 (9)	−0.0037 (10)	−0.0015 (9)
C8	0.0219 (12)	0.0220 (12)	0.0248 (12)	0.0015 (9)	−0.0009 (9)	0.0023 (9)
C9	0.0265 (13)	0.0255 (13)	0.0390 (15)	−0.0012 (10)	0.0022 (11)	−0.0092 (10)
C10	0.0248 (13)	0.0322 (15)	0.0378 (15)	−0.0050 (11)	0.0007 (11)	−0.0071 (11)
C11	0.0248 (13)	0.0253 (13)	0.0282 (13)	0.0026 (10)	0.0021 (10)	0.0007 (10)
C12	0.0306 (13)	0.0235 (13)	0.0282 (13)	0.0012 (10)	0.0028 (10)	−0.0051 (9)
C13	0.0255 (12)	0.0226 (12)	0.0274 (13)	−0.0024 (10)	−0.0006 (10)	−0.0014 (9)
C14	0.0299 (13)	0.0246 (13)	0.0332 (14)	0.0015 (10)	0.0017 (11)	−0.0005 (10)

### Geometric parameters (Å, º)

**Table:**

N1—C7	1.357 (3)	C4—C5	1.399 (3)
N1—C5	1.375 (3)	C4—H4	0.9300
N1—H1	0.8600	C5—C6	1.406 (3)
N2—C7	1.325 (3)	C7—C8	1.471 (3)
N2—C6	1.395 (3)	C8—C13	1.393 (3)
F1—C14	1.332 (3)	C8—C9	1.393 (3)
F2—C14	1.318 (3)	C9—C10	1.379 (4)
F3—C14	1.342 (3)	C9—H9	0.9300
C1—C2	1.377 (3)	C10—C11	1.386 (3)
C1—C6	1.395 (3)	C10—H10	0.9300
C1—H1A	0.9300	C11—C12	1.380 (3)
C2—C3	1.405 (4)	C11—C14	1.498 (3)
C2—H2	0.9300	C12—C13	1.391 (3)
C3—C4	1.380 (3)	C12—H12	0.9300
C3—H3	0.9300	C13—H13	0.9300
			
C7—N1—C5	107.02 (19)	C13—C8—C9	119.5 (2)
C7—N1—H1	126.5	C13—C8—C7	119.8 (2)
C5—N1—H1	126.5	C9—C8—C7	120.7 (2)
C7—N2—C6	104.73 (18)	C10—C9—C8	120.2 (2)
C2—C1—C6	117.9 (2)	C10—C9—H9	119.9
C2—C1—H1A	121.0	C8—C9—H9	119.9
C6—C1—H1A	121.0	C9—C10—C11	119.9 (2)
C1—C2—C3	121.7 (2)	C9—C10—H10	120.1
C1—C2—H2	119.2	C11—C10—H10	120.1
C3—C2—H2	119.2	C12—C11—C10	120.6 (2)
C4—C3—C2	121.5 (2)	C12—C11—C14	121.2 (2)
C4—C3—H3	119.3	C10—C11—C14	118.3 (2)
C2—C3—H3	119.3	C11—C12—C13	119.8 (2)
C3—C4—C5	116.7 (2)	C11—C12—H12	120.1
C3—C4—H4	121.6	C13—C12—H12	120.1
C5—C4—H4	121.6	C12—C13—C8	120.0 (2)
N1—C5—C4	132.1 (2)	C12—C13—H13	120.0
N1—C5—C6	105.74 (19)	C8—C13—H13	120.0
C4—C5—C6	122.2 (2)	F2—C14—F1	107.5 (2)
N2—C6—C1	130.7 (2)	F2—C14—F3	106.0 (2)
N2—C6—C5	109.3 (2)	F1—C14—F3	105.1 (2)
C1—C6—C5	120.0 (2)	F2—C14—C11	113.0 (2)
N2—C7—N1	113.2 (2)	F1—C14—C11	111.8 (2)
N2—C7—C8	124.5 (2)	F3—C14—C11	112.9 (2)
N1—C7—C8	122.3 (2)		
			
C6—C1—C2—C3	−1.3 (4)	N1—C7—C8—C13	150.3 (2)
C1—C2—C3—C4	−0.1 (4)	N2—C7—C8—C9	149.8 (2)
C2—C3—C4—C5	1.2 (3)	N1—C7—C8—C9	−30.0 (3)
C7—N1—C5—C4	−179.0 (2)	C13—C8—C9—C10	−0.1 (4)
C7—N1—C5—C6	−0.3 (2)	C7—C8—C9—C10	−179.7 (2)
C3—C4—C5—N1	177.6 (2)	C8—C9—C10—C11	0.0 (4)
C3—C4—C5—C6	−1.0 (3)	C9—C10—C11—C12	0.3 (4)
C7—N2—C6—C1	179.2 (2)	C9—C10—C11—C14	−179.7 (2)
C7—N2—C6—C5	−0.6 (2)	C10—C11—C12—C13	−0.4 (4)
C2—C1—C6—N2	−178.2 (2)	C14—C11—C12—C13	179.6 (2)
C2—C1—C6—C5	1.5 (3)	C11—C12—C13—C8	0.3 (4)
N1—C5—C6—N2	0.5 (2)	C9—C8—C13—C12	−0.1 (4)
C4—C5—C6—N2	179.4 (2)	C7—C8—C13—C12	179.6 (2)
N1—C5—C6—C1	−179.2 (2)	C12—C11—C14—F2	106.1 (3)
C4—C5—C6—C1	−0.3 (3)	C10—C11—C14—F2	−73.9 (3)
C6—N2—C7—N1	0.4 (3)	C12—C11—C14—F1	−132.4 (3)
C6—N2—C7—C8	−179.4 (2)	C10—C11—C14—F1	47.6 (3)
C5—N1—C7—N2	−0.1 (3)	C12—C11—C14—F3	−14.1 (3)
C5—N1—C7—C8	179.7 (2)	C10—C11—C14—F3	165.8 (2)
N2—C7—C8—C13	−29.9 (3)		

### Hydrogen-bond geometry (Å, º)

Cg is the centroid of the N1/C5/C6/N2/C7 ring.

**Table:**

*D*—H···*A*	*D*—H	H···*A*	*D*···*A*	*D*—H···*A*
N1—H1···N2^i^	0.86	2.07	2.914 (3)	165
C12—H12···F1^ii^	0.93	2.57	3.374 (3)	144
C13—H13···F3^iii^	0.93	2.55	3.275 (4)	134
C2—H2···*Cg*^iv^	0.93	2.94	3.700 (3)	140

Symmetry codes: (i) −*x*+3/2, *y*−1/2, *z*; (ii) −*x*+1/2, *y*+1/2, *z*; (iii) *x*+1/2, −*y*+3/2, −*z*; (iv) *x*+1/2, *y*, −*z*+1/2.
